# Thermal and Concentration Stratifications Effects in Radiative Flow of Jeffrey Fluid over a Stretching Sheet

**DOI:** 10.1371/journal.pone.0107858

**Published:** 2014-10-02

**Authors:** T. Hayat, Tariq Hussain, S. A. Shehzad, A. Alsaedi

**Affiliations:** 1 Department of Mathematics, Quaid-i-Azam University, Islamabad, Pakistan; 2 Nonlinear Analysis and Applied Mathematics (NAAM) Research Group, Department of Mathematics, Faculty of Science, King Abdulaziz University, Jeddah, Saudi Arabia; 3 Department of Mathematics, Faculty of Computing, Mohammad Ali Jinnah University, Islamabad Campus, Islamabad, Pakistan; 4 Department of Mathematics, Comsats Institute of Information Technology, Sahiwal, Pakistan; Tsinghua University, China

## Abstract

In this article we investigate the heat and mass transfer analysis in mixed convective radiative flow of Jeffrey fluid over a moving surface. The effects of thermal and concentration stratifications are also taken into consideration. Rosseland's approximations are utilized for thermal radiation. The nonlinear boundary layer partial differential equations are converted into nonlinear ordinary differential equations via suitable dimensionless variables. The solutions of nonlinear ordinary differential equations are developed by homotopic procedure. Convergence of homotopic solutions is examined graphically and numerically. Graphical results of dimensionless velocity, temperature and concentration are presented and discussed in detail. Values of the skin-friction coefficient, the local Nusselt and the local Sherwood numbers are analyzed numerically. Temperature and concentration profiles are decreased when the values of thermal and concentration stratifications parameters increase. Larger values of radiation parameter lead to the higher temperature and thicker thermal boundary layer thickness.

## Introduction

The boundary layer flow of non-Newtonian fluids gains a special attention of the researchers because of its wide occurrence in the industrial and engineering processes. The most commonly involved fluids in industry and technology are categorized as non-Newtonian. Many of the materials used in biological sciences, chemical and petroleum industries, geophysics etc. are also known as the non-Newtonian fluids. The non-Newtonian fluids are further divided into three main classes namely differential, rate and integral types. The simplest subclass of non-Newtonian fluids is the rate type fluids. The present study involves the Jeffrey fluid model which falls into the category of rate type non-Newtonian fluids. This fluid model exhibits the properties of ratio of relaxation to retardation times and retardation time. This model is very popular amongst the investigators. Few studies regarding Jeffrey fluid model are mentioned in the references [1]–[5].

The better cooling rate in the manufacturing processes is very essential for the best quality final product. For such processes, a controlled cooling system is required. An electrically polymeric liquid seems to be a good candidate for such applications of polymer and metallurgy because here the flow can be controlled by an applied magnetic field. Further the magnetohydrodynamic (MHD) flows are quite prominent in MHD power generating systems, cooling of nuclear reactors, plasma studies, geothermal energy extraction and many others. Interesting investigations on MHD flows can be seen in the references [6]–[11]. The thermal radiation effects have pivotal role in the industrial and engineering processes. Such processes are performed at very high temperature under various non-isothermal conditions and in situations where convective heat transfer coefficients are smaller. The radiative heat transfer can be used in hypersonic flights, model of pertinent equipment, nuclear power plants, nuclear reactors, gas turbines, space vehicles etc. [12]–[15].

Influence of stratification is an important aspect in heat and mass transfer analysis. The formation or deposition of the layers is known as the stratification. This phenomenon occurs due to the change in temperature or concentration, or variations in both, or presence of various fluids or different densities. It is quite important and interesting to examine the effects of combined stratifications (thermal and concentration stratifications) in mixed convective flow past a surface when heat and mass transfer analysis is performed simultaneously. Investigation of doubly stratified flows is a subject of special attention nowadays because of its broad range of applications in industrial and engineering processes. Few practical examples of these applications include heat rejection into the environment such as rivers, seas and lakes, thermal energy storage systems like solar ponds, mixture in industrial, food and manufacturing processing, density stratification of the atmosphere etc. Having all such applications in view, Hayat et al. [16] provided an analysis to examine the thermal stratification effects in mixed convective flow of Maxwell fluid over a stretching surface. Simultaneous effects of thermal stratification and thermal radiation in stretched flow of thixotropic fluid are discussed by Shehzad et al. [17]. Ibrahim and Makinde [18] considered the effects of double stratifications in mixed convection flow of nanofluid past a vertical plate. Srinivasacharya and Upender [19] investigated the doubly stratified flow of micropolar fluid in the presence of an applied magnetic field. Soret and Dufour effects on doubly stratified flow of viscous fluid in a porous medium are studied by Srinivasacharya and Surender [20].

Here our main theme is to study the influences of thermal and concentration stratifications in mixed convection flow of Jeffrey fluid over a stretching sheet. Heat and mass transfer characteristics are encountered. Further, we considered the thermal radiation effect. Mathematical modelling is presented subject to boundary layer assumptions and Roseland's approximation. The governing nonlinear flow model is solved and homotopic solutions [21]–[35] of dimensionless velocity, temperature and concentration are presented. Physical quantities for various parameters of interest are examined. To our knowledge such analysis is not yet reported.

## Governing Problems

**Figure 1:**
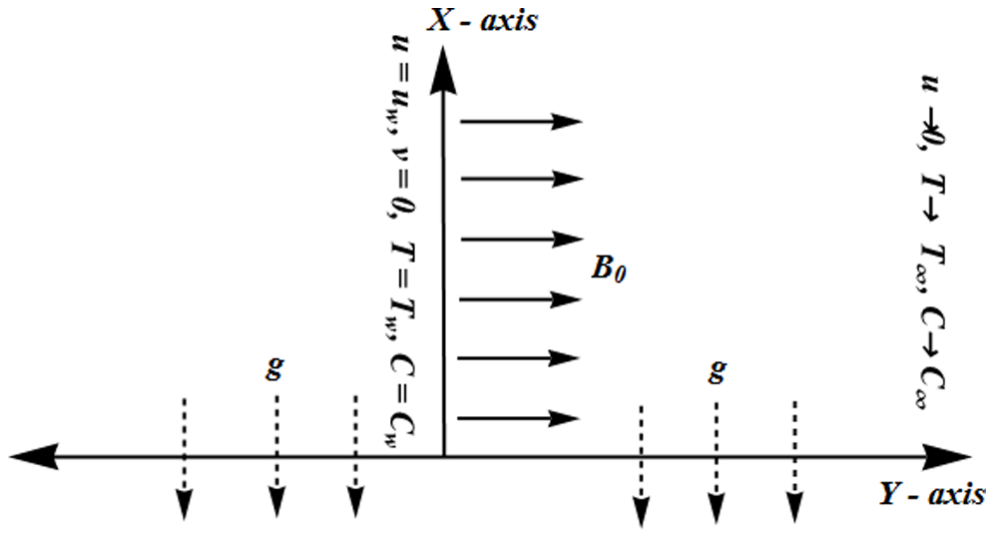
Physical model.



We consider the mixed convection flow of an incompressible Jeffrey fluid over a stretching surface. Thermal and concentration stratifications are taken into account in the presence of thermal radiation. The vertical surface has temperature 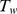 and concentration 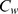 and further 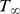 and 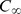 are the temperature and concentration of ambient fluid. The 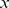 and 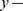 axes are chosen along and normal to the surface. The magnetic field of strength *B_0_* is applied normal to the flow direction (see Fig. 1). The effects of induced magnetic field are neglected due to the low magnetic Reynolds number. The governing partial differential equations under boundary layer assumptions are given below [4], [14]:

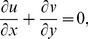
(1)

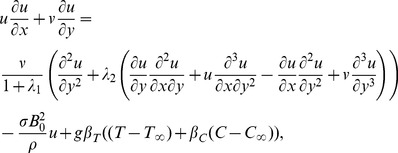
(2)

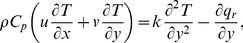
(3)

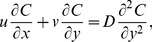
(4)where 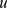 and 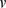 denote the velocity components in the 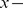 and 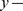 directions, 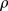 the fluid density, 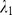 the ratio of relaxation to retardation times, 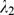 the retardation time, 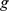 the gravitational acceleration, 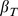 the thermal expansion coefficient, 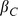 the concentration expansion coefficient, 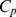 the specific heat at constant pressure, 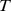 the fluid temperature, 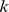 the thermal conductivity of fluid, 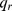 the radiative heat flux, 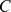 the fluid concentration and 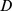 the diffusion coefficient.

The subjected boundary conditions are [18]:
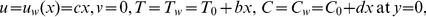
(5)

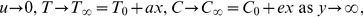
(6)in which 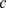 is the stretching rate, 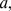 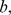 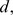 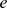 are dimensional constants and 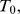 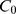 are the reference temperature and reference concentration, respectively.

The radiative flux is accounted by employing the Rosseland assumption in the energy equation [12], [15]:
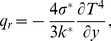
(7)in which 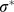 the Stefan-Boltzmann constant and 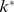 the mean absorption coefficient. Further, the differences of temperature within the flow is assumed to be small such that 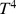 may be expressed as a linear function of temperature. Expansion of 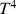 about 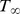 via Taylor's series and ignoring higher order terms, we have



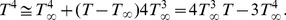
(8)By employing Eqs. (7) and (8), Eq. (3) has the form
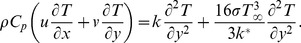
(9)


Setting
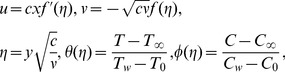
(10)
equation (1) is satisfied automatically and reduced forms of Eqs. (7)–(10) and (13) are



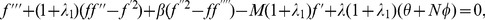
(11)

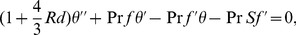
(12)

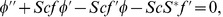
(13)and the boundary conditions in dimensionless form has the following form



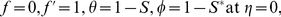
(14)

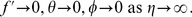
(15)


Here 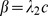 is the Deborah number, 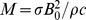 the magnetic parameter, 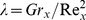 the thermal buoyancy parameter with 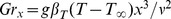 the local Grashof number and 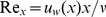 the local Reynolds number, 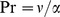 the Prandtl number, 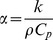 the thermal diffusivity, 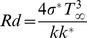 the thermal radiation parameter, 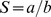 the thermal stratification parameter, 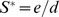 the concentration stratification parameter, 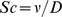 the Schmidt number and 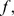 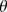 and 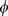 the dimensionless velocity, temperature and concentration, respectively.

The skin friction coefficient, the local Nusselt number and the local Sherwood number are
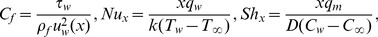
(16)where 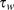 is the shear stress along the stretching surface, 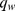 is the surface heat flux and 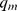 is the surface mass flux. The local skin-friction coefficient, local Nusselt and local Sherwood numbers in dimensionless forms are given below:
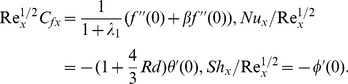
(17)


## Development of the Series Solutions

To develop the homotopic procedure [21]–[23], we choose the initial guesses and operators in the forms given below:
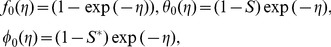
(18)

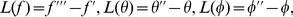
(19)with
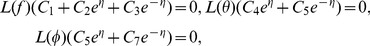
(20)where 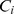 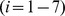 are the arbitrary constants. The zeroth order deformation equations together with the boundary conditions are



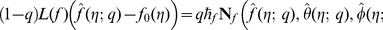
(21)




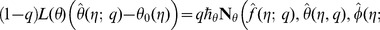
(22)

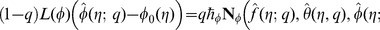
(23)

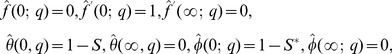
(24)

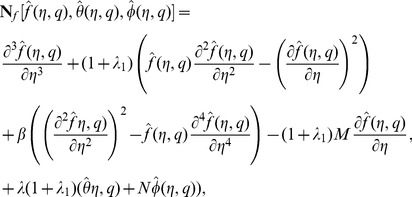
(25)

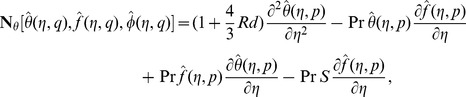
(26)

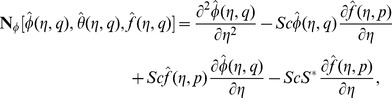
(27)where 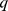 is an embedding parameter, 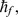 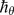 and 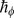 the non-zero auxiliary parameters and 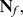 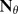 and 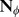 the nonlinear operators. For 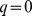 and 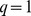 one has



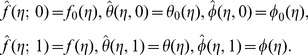
(28)


When variation of 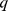 is taken into account from 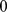 to 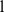 then 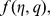 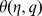 and 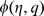 vary from 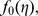 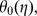 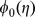 to 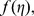 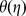 and 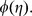 We expand 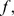 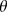 and 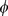 in the following forms [24]–[26]:



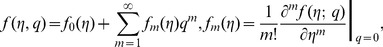
(29)

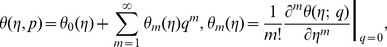
(30)

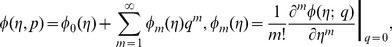
(31)where the convergence of above series strongly depends upon 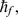 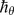 and 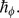 Considering that 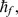 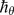 and 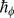 are selected properly such that Eqs. (29)–(31) converge for 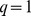 and thus [27], [28]:



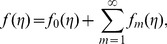
(32)

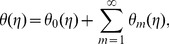
(33)

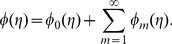
(34)


The general solutions are derived as follows:



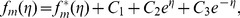
(35)




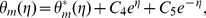
(36)

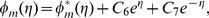
(37)where 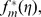 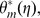 and 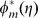 are the special solutions.

## Analysis and Discussion

**Figure 2:**
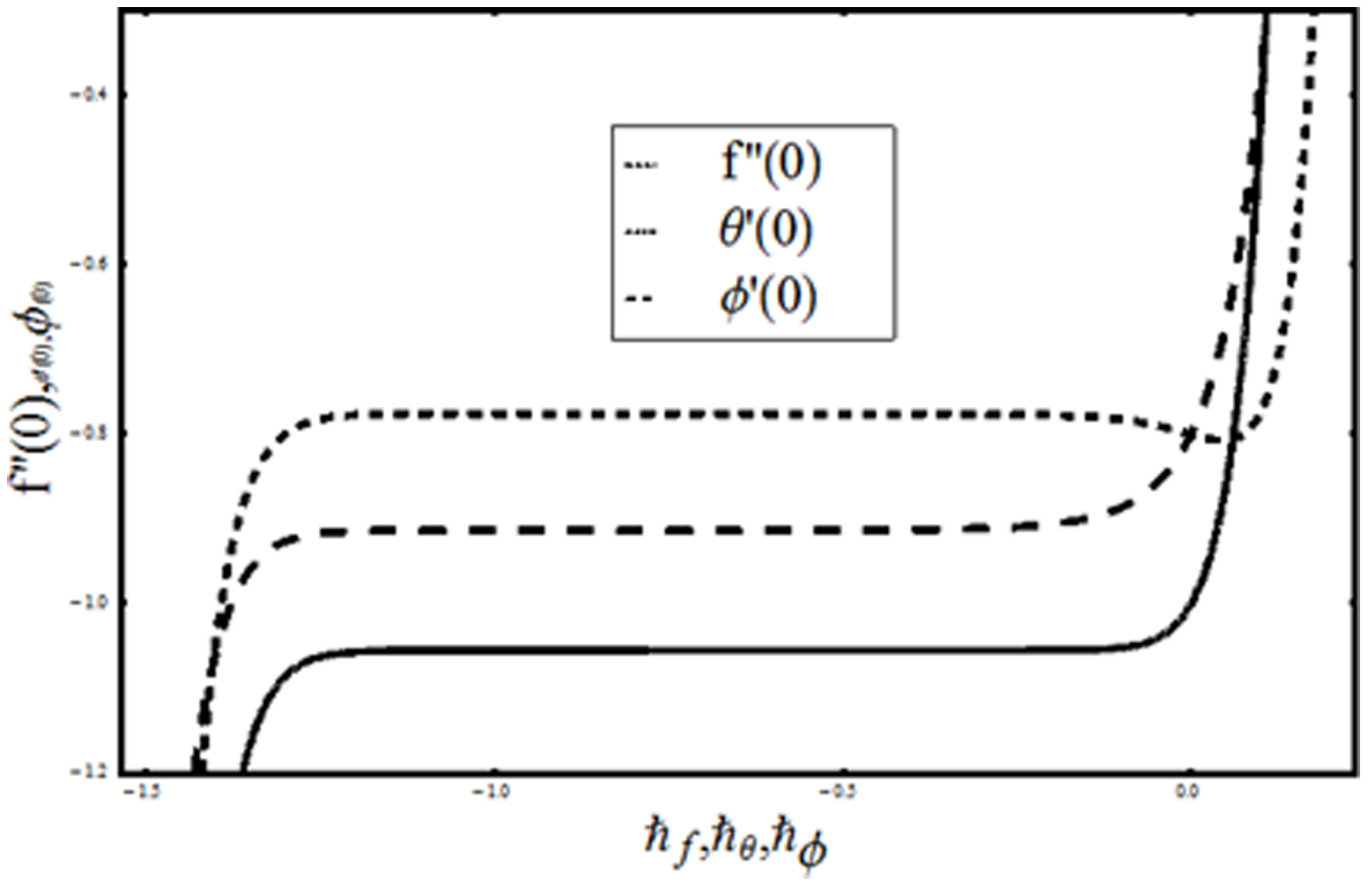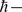 curves of functions 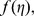 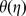 and 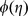 at 17th-order of deformations when 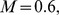 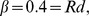 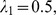 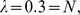 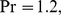 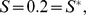 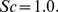

The coupled nonlinear ordinary differential equations are solved via homotopy analysis method. The convergence of derived homotopic solutions depend on the suitable values of auxiliary parameters 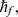 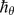 and 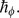 Hence the 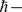 curves of functions 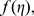 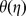 and 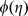 are drawn at 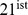 -order of approximations to choose the admissible values of 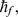 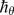 and 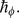 From Fig. 2 we have seen that the range of admissible values of 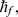 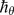 and 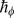 are 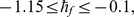 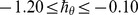 and 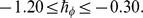 Table 1 also shows that the developed homotopic solutions are convergent in the whole region of 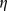 when 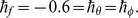


**Table 1:** Convergence of homotopy solution for different order of approximations when 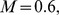 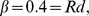 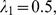 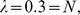 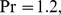 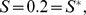 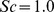 and 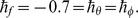

Order of approximation	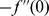	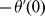	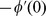
01	1.060200	0.790667	0.870000
10	1.055105	0.776083	0.913029
17	1.055116	0.776004	0.913502
24	1.055118	0.775996	0.913543
28	1.055118	0.775996	0.913547
35	1.055118	0.775996	0.913547
40	1.055118	0.775996	0.913547

**Figure 3:**
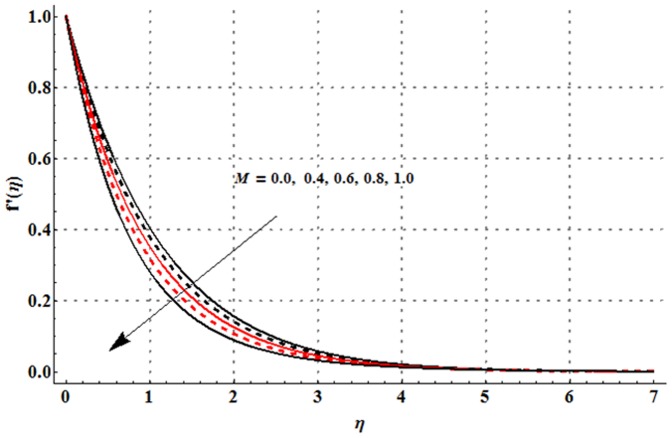
Influence of 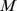 on the velocity profile 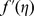 when 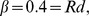 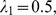 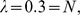 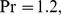 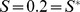 and 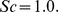

**Figure 4:**
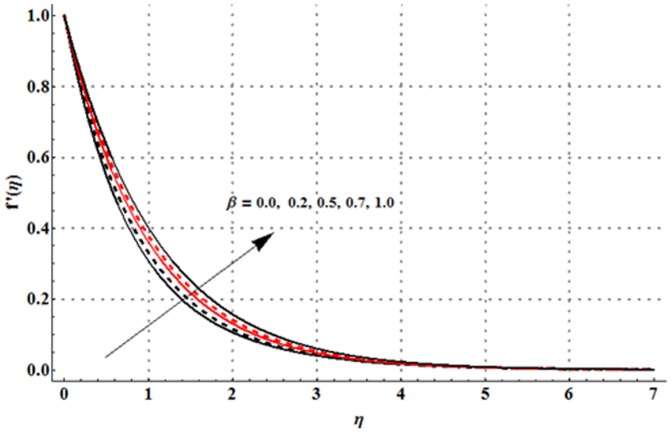
Influence of 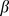 on the velocity profile 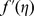 when 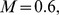 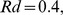 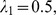 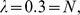 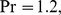 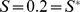 and 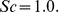

**Figure 5:**
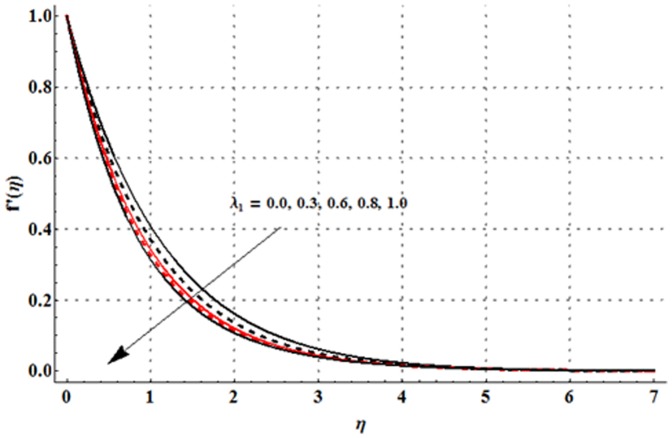
Influence of 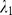 on the velocity profile 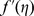 when 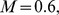 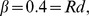 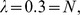 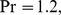 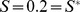 and 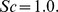

**Figure 6:**
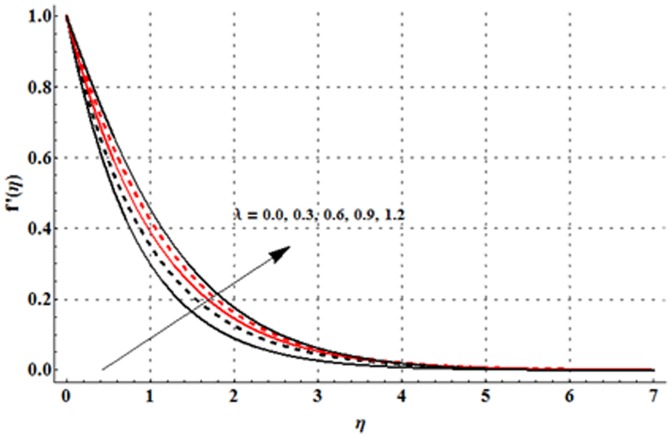
Influence of 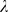 on the velocity profile 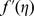 when 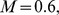 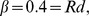 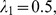 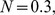 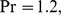 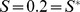 and 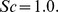

**Figure 7:**
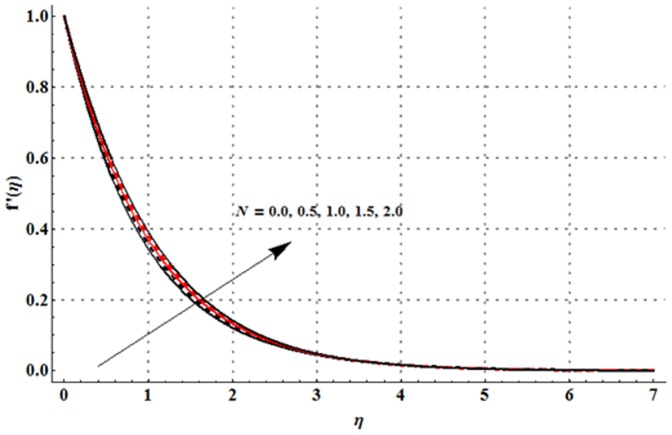
Influence of 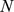 on the velocity profile 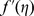 when 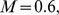 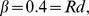 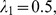 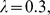 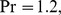 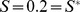 and 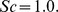

**Figure 8:**
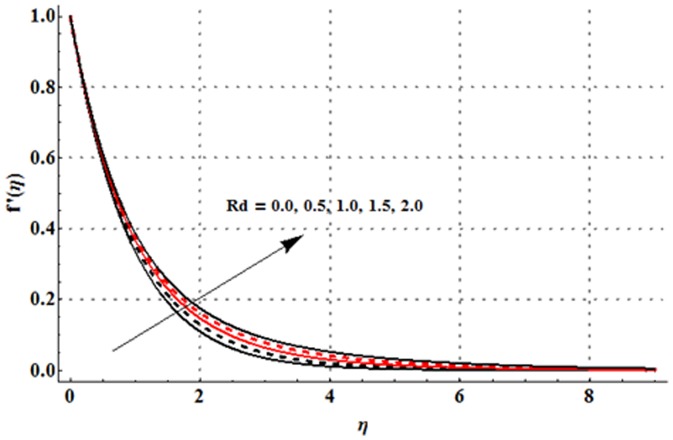
Influence of 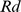 on the velocity profile 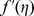 when 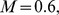 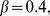 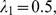 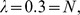 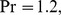 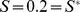 and 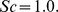

The dimensionless velocity profile 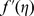 for different values of magnetic parameter 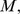 Deborah number 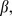 ratio of relaxation to retardation times 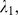 thermal buoyancy parameter 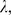 concentration buoyancy parameter 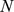 and radiation parameter 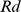 is sketched in the Figs. 3–8. It is noticed from Fig. 3 that the velocity profile and momentum boundary layer thickness is reduced when larger values of magnetic parameter are used. Here the magnetic parameter involves the Lorentz force. Lorentz force has an ability to resist the fluid flow. Such resistance in fluid flow leads to a reduction in the velocity profile. From Fig. 4 it is observed that larger Deborah number shows higher velocity and thicker momentum boundary layer thickness. From the definition of Deborah number, one can see that the Deborah number is directly proportional to the retardation time. Larger Deborah number has higher retardation time. Such higher retardation time gives rise to the fluid flow due to which the velocity profile is enhanced. Fig. 5 illustrates the impact of ratio of relaxation to retardation times on the velocity field. This Fig. shows that the velocity and its related boundary layer thickness are decreasing functions of ratio of relaxation to retardation times. Fig. 6 depicts that an increase in thermal buoyancy parameter leads to an increase in the velocity profile. Thermal buoyancy parameter depends on the buoyancy force. Larger buoyancy parameter has stronger buoyancy force. Such stronger buoyancy force acts as an agent and causes to an increase in the fluid velocity. Fig. 7 elucidates that both velocity profile and its related momentum boundary layer thickness are enhanced with an increase in the concentration buoyancy parameter. The change in velocity distribution function for various values of radiation parameter is examined in Fig. 8. Here we observed that the velocity distribution function is increased when we increase the values of radiation parameter.

**Figure 9:**
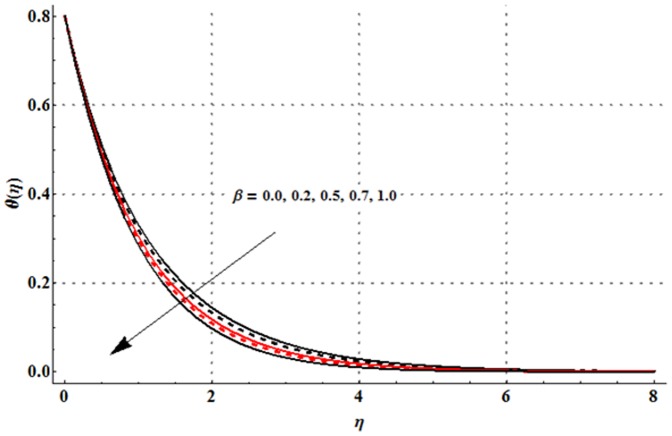
Influence of 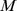 on the temperature profile 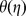 when 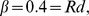 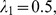 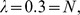 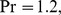 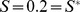 and 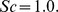

**Figure 10:**
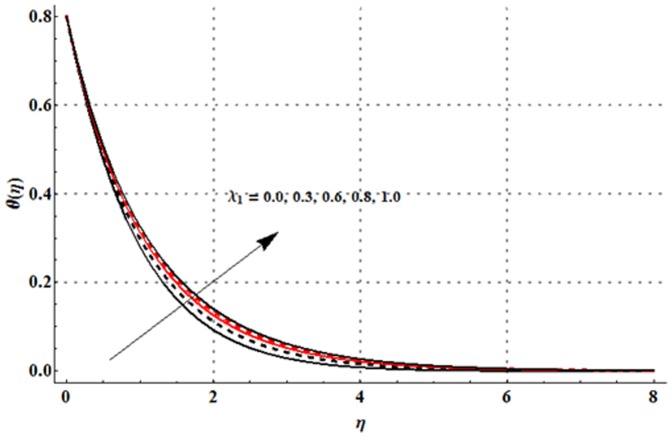
Influence of 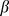 on the temperature profile 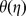 when 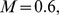 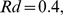 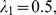 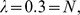 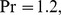 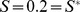 and 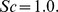

**Figure 11:**
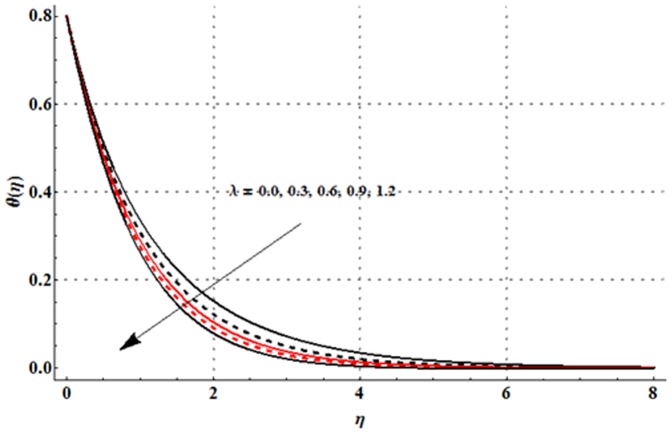
Influence of 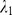 on the temperature profile 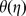 when 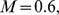 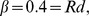 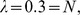 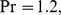 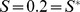 and 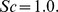

**Figure 12:**
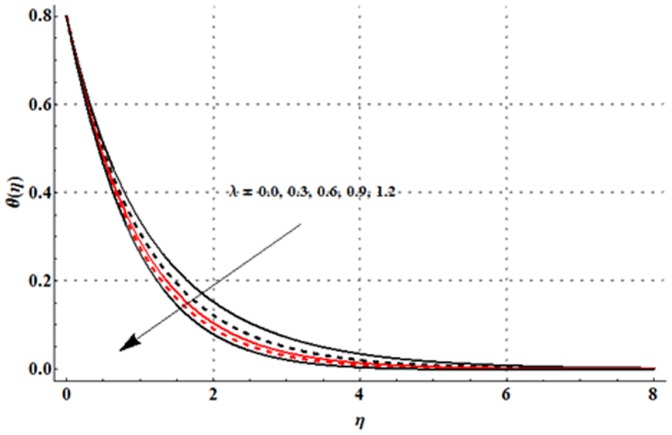
Influence of 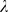 on the temperature profile 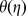 when 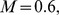 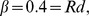 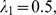 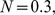 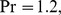 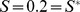 and 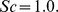

**Figure 13:**
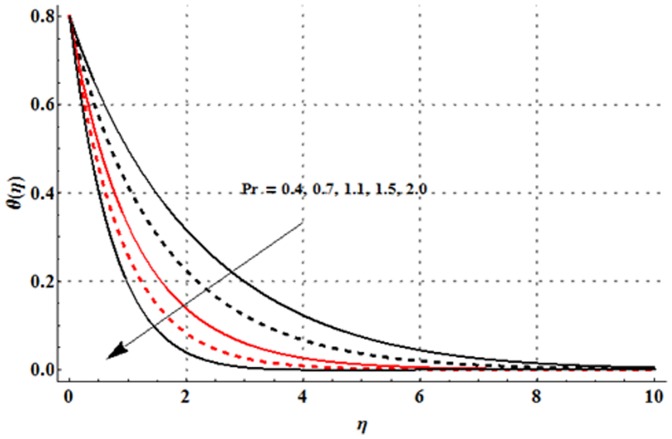
Influence of 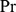 on the temperature profile 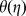 when 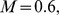 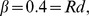 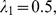 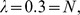 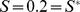 and 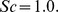

**Figure 14:**
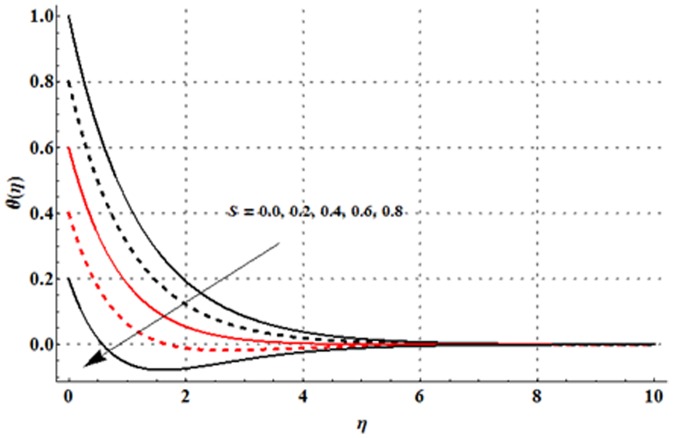
Influence of 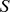 on the temperature profile 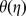 when 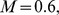 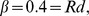 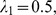 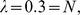 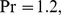 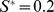 and 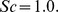

**Figure 15:**
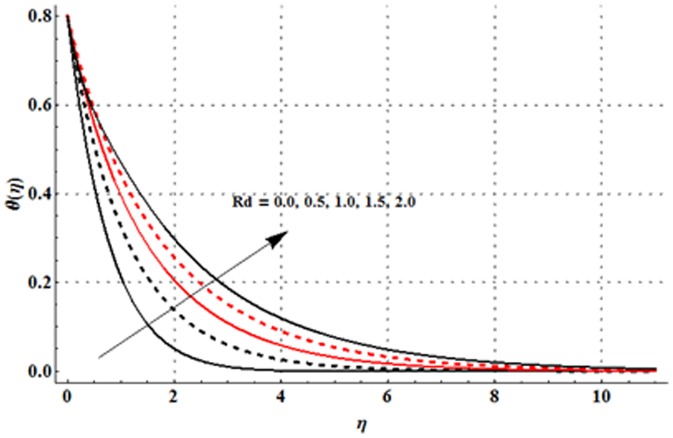
Influence of 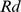 on the temperature profile 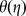 when 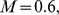 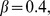 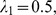 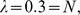 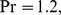 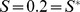 and 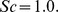

The variations in the non-dimensional temperature distribution function 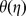 correspond to different values of magnetic parameter 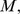 Deborah number 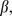 ratio of relaxation to retardation times 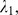 thermal buoyancy parameter 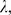 Prandtl number 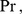 thermal stratification parameter 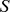 and radiation parameter 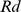 are examined in the Figs. 9–15. From Fig. 9 it is seen that the temperature profile and thermal boundary layer thickness are enhanced for the larger magnetic parameter. Here stronger Lorentz force corresponds to the larger magnetic parameter. This stronger Lorentz force has an ability to increase the temperature. Figs. 10 and 11 depict that the Deborah number and ratio of relaxation to retardation times have quite reverse effects on the temperature field and thermal boundary layer thickness. Temperature is decreased with an increase in the Deborah number but an enhancement in the temperature is observed for larger ratio of relaxation to retardation times. Fig. 12 illustrates that an increase in the thermal buoyancy parameter leads to a reduction in the temperature profile and thermal boundary layer thickness. From Fig. 13 we observed that lower temperature and thinner thermal boundary layer thickness correspond to an increase in the Prandtl number. Prandtl number is the ratio of momentum to thermal diffusivities. An enhancement in the Prandtl number implies to higher momentum diffusivity and lower thermal diffusivity. Such variation in momentum and thermal diffusivities shows a reduction in the temperature profile and thermal boundary layer thickness. Fig. 14 is sketched for temperature field when different values of thermal stratification parameter are taken into account. We have seen that the temperature profile is reduced when we increase the values of thermal stratification parameter. It is also noticed that the case of prescribed surface temperature is obtained when 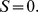 Physically, the difference between the surface temperature and ambient temperature is decreased when larger values of thermal stratification parameter are used. This change in surface and ambient temperatures leads to a decrease in the temperature profile. From Fig. 15 we noticed that higher temperature and thicker thermal boundary layer thickness correspond to the larger radiation parameter. Here larger radiation parameter gives more heat to fluid due to which the temperature profile is enhanced.

**Figure 16:**
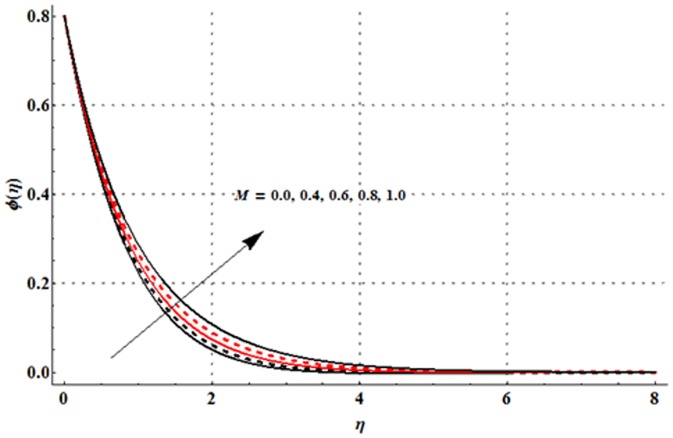
Influence of 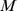 on the concentration profile 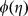 when 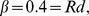 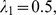 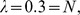 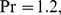 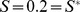 and 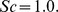

**Figure 17:**
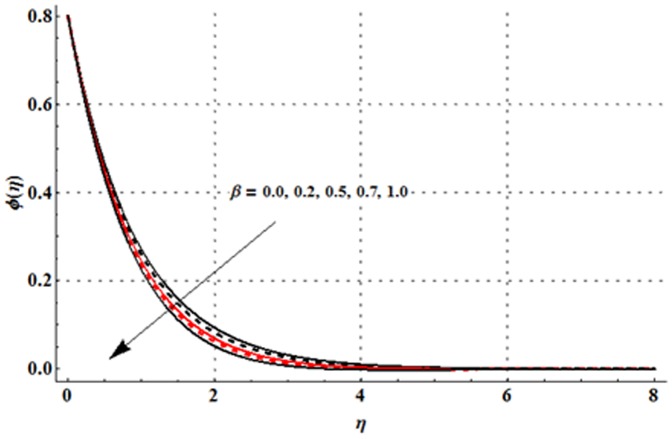
Influence of 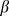 on the concentration profile 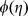 when 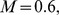 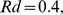 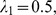 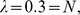 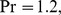 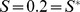 and 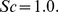

**Figure 18:**
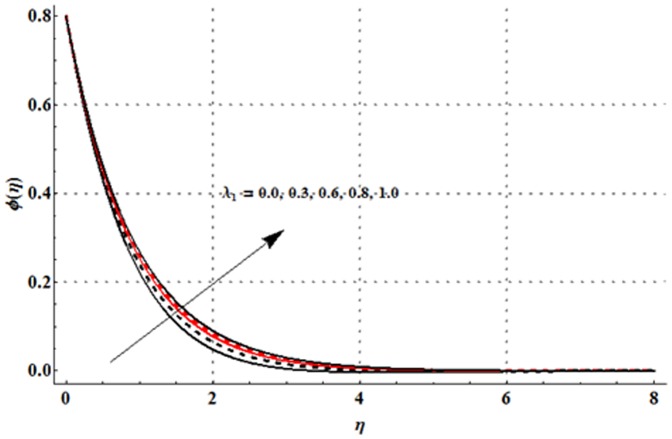
Influence of 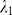 on the concentration profile 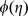 when 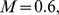 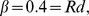 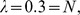 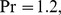 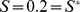 and 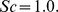

**Figure 19:**
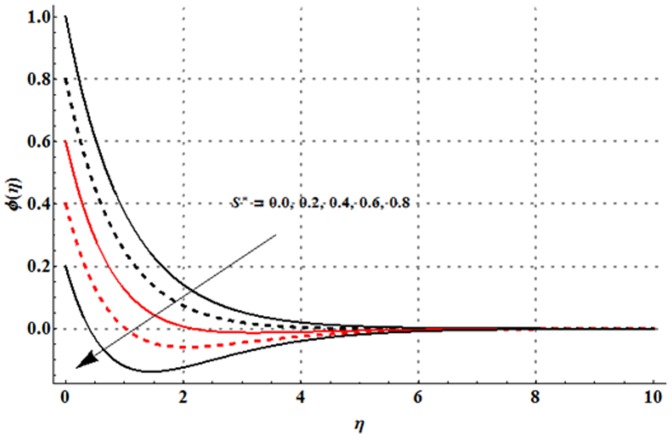
Influence of 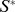 on the concentration profile 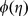 when 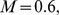 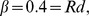 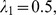 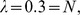 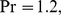 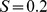 and 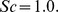

**Figure 20:**
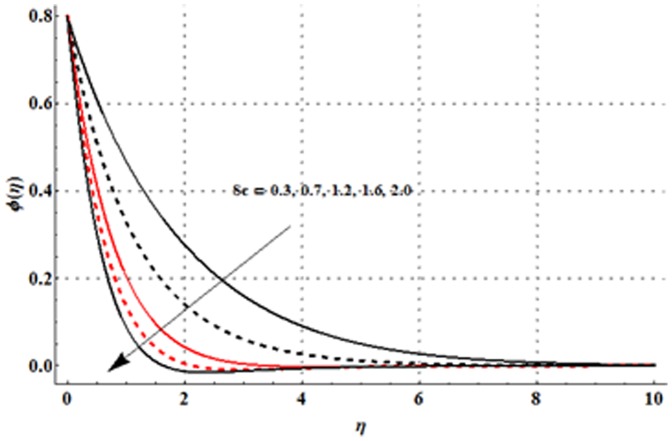
Influence of 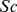 on the concentration profile 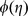 when 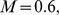 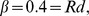 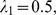 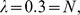 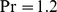 and 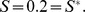

The effects of magnetic parameter 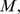 Deborah number 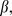 ratio of relaxation to retardation times 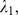 concentration stratification parameter 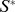 and Schmidt number 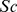 on the concentration field 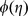 are shown in the Figs. 16–20. Fig. 16 elucidates that concentration profile and its associated boundary layer thickness are increased with an increase in the magnetic parameter. From Figs. 17 and 18, we observed that the concentration is decreased for larger Deborah number but the larger values of ratio of relaxation to retardation times give rise to the concentration field. Impact of concentration stratification parameter on the concentration profile is examined in Fig. 19. From this Fig. it is observed that the concentration profile is reduced with an increase in the concentration stratification parameter. Further prescribed surface concentration case is achieved when we use 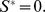 An increase in Schmidt number leads to a reduction in the concentration profile and its related boundary layer thickness (see Fig. 20).

The numerical values of 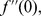 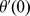 and 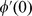 at different order of HAM approximations are analyzed in Table 1 when 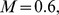 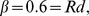 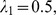 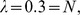 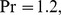 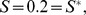 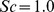 and 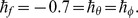 From this Table it is noticed that the values of 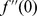 and 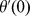 start to repeat from 24th-order of deformations. On the other hand the values of 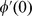 converge from 28th-order of approximations. Table 2 presents the numerical values of skin-friction coefficient for different values of 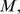 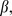 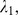 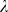 and 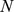 when 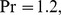 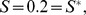 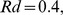 and 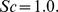 It is observed that the values of skin-friction coefficient are larger when we increase the values of 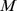 and 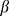 but these values are smaller for larger 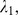 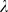 and 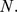 Table 3 is computed to examine the values of skin-friction coefficient for different values of 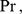 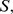 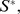 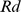 and 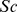 when 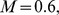 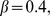 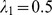 and 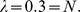 This Table shows that the values of skin-friction coefficient are increased with an increase in 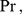 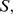 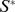 and 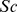 but a decrease is noticed for the larger 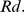 Numerical values of local Nusselt and Sherwood numbers for various values of 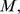 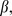 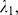 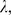 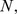 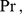 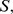 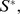 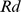 and 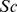 are observed in the Tables 4 and 5. From these Tables we have seen that the values of local Nusselt number are larger in comparison to the values of local Sherwood number when we used the values of 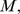 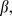 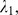 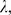 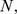 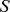 and 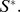


**Table 2:** Values of skin-friction coefficient 
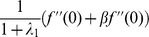
 for different values of 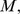 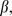 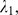 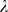 and 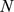 when 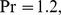 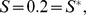 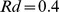 and 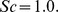

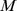	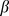	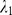	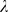	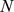	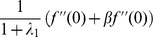
0.0	0.4	0.5	0.3	0.3	0.82197
0.4					0.89699
0.8					1.09849
0.6	0.0	0.5	0.3	0.3	0.81634
	0.3				0.94515
	0.7				1.09609
0.6	0.4	0.0	0.3	0.3	1.23185
		0.6			0.95028
		1.0			0.84010
0.6	0.4	0.5	0.0	0.3	1.12665
			0.4		0.81119
			0.7		0.94018
0.6	0.4	0.5	0.3	0.0	1.01394
				0.4	0.97514
				0.7	0.94646

**Table 3:** Values of skin-friction coefficient 
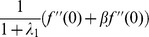
 for different values of 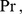 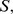 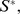 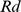 and 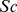 when 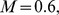 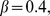 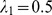 and 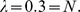

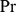	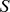	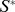	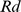	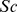	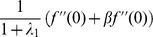
0.7	0.2	0.2	0.4	1.0	0.96396
1.0					0.97756
1.3					0.98797
1.2	0.0	0.2	0.4	1.0	0.94859
	0.5				1.03868
	0.7				1.07436
1.2	0.2	0.0	0.4	1.0	0.97432
		0.5			1.00044
		0.7			1.01086
1.2	0.2	0.2	0.0	1.0	1.00186
			0.5		0.98145
			0.8		0.96839
1.2	0.2	0.2	0.4	0.8	0.98212
				2.0	0.99263
				2.5	0.99495

**Table 4:** Values of local Nusselt number 
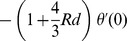
 and local Sherwood number 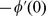 for different values of 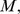 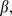 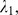 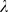 and 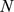 when 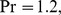 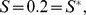 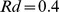 and 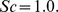

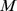	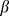	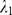	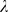	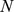	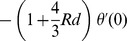	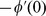
0.0	0.4	0.5	0.3	0.3	1.24653	0.95218
0.4					1.22027	0.93431
0.8					1.15112	0.88693
0.6	0.0	0.5	0.3	0.3	1.14034	0.87924
	0.3				1.17885	0.90598
	0.7				1.21882	0.93328
0.6	0.4	0.0	0.3	0.3	1.25054	0.95473
		0.6			1.18031	0.90697
		1.0			1.14782	0.88445
0.6	0.4	0.5	0.0	0.3	1.12659	0.87086
			0.4		1.20514	0.92399
			0.7		1.24331	0.95029
0.6	0.4	0.5	0.3	0.0	1.18090	0.90734
				0.4	1.19273	0.91552
				0.7	1.20103	0.92126

**Table 5:** Values of local Nusselt number 
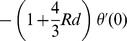
 and local Sherwood number 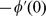 for different values of 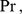 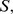 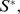 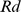 and 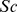 when 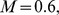 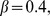 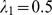 and 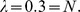

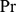	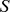	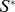	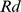	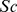	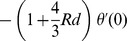	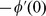
0.7	0.2	0.2	0.4	1.0	0.84401	0.93252
1.0					1.05959	0.91966
1.3					1.25180	0.91100
1.2	0.0	0.2	0.4	1.0	1.31047	0.92744
	0.5				1.00330	0.89150
	0.7				0.87521	0.87585
1.2	0.2	0.0	0.4	1.0	1.19544	0.99065
		0.5			1.18137	0.79709
		0.7			1.17562	0.71897
1.2	0.2	0.2	0.0	1.0	1.01578	0.90118
			0.5		1.22660	0.91628
			0.8		1.32621	0.92373
1.2	0.2	0.2	0.4	0.8	1.19275	0.78950
				2.0	1.18395	1.40976
				2.5	1.18275	1.61211

## Conclusions

We examined the effects of thermal and concentration stratifications in mixed convective radiative flow of Jeffrey fluid in this attempt. The main observations that we found in this investigation are as follows:

- We have to compute 28th-order of HAM deformations for the convergent solutions.
- Deborah number 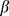 and ratio of relaxation to retardation times have reverse effects on the velocity profile 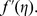

- The effects of thermal buoyancy parameters on the velocity field 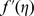 are more pronounced in comparison to concentration buoyancy parameter.
- An increase in thermal stratification parameter 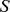 leads to a reduction in the temperature field and thermal boundary layer thickness.
- The temperature profile and thermal boundary layer thickness are enhanced when radiation parameter 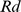 is increased.
- The concentration field and its associated boundary layer thickness are decreasing functions of concentration stratification parameter 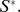

- Numerical values of skin-friction coefficient are increased by increasing 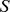 and 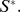

- The larger values of 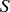 and 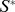 correspond to the lower values of local Nusselt and local Sherwood numbers.
